# Effects of pre- and post-exercise intake of performance supplements on body composition, circumferences, and muscle strength in trained men during 6 weeks of resistance training

**DOI:** 10.1186/1550-2783-8-S1-P23

**Published:** 2011-11-07

**Authors:** Michael J Ormsbee, David Thomas, William K Mandler, Amber W Kinsey, Colin J Riley, Lynn B Panton, Jeong-Su Kim

**Affiliations:** 1 Department of Nutrition, Food, and Exercise Sciences. The Florida State University, Tallahassee, FL 32306, USA

## Background

Resistance training (RT) enhances muscle protein synthesis and increases muscle strength and hypertrophy. Protein and amino acid supplements have been shown to augment the physiological improvements associated with RT such as improved body composition, muscular strength, and hypertrophy while suppressing exercise-induced proteolysis. Supplements that also contain creatine and caffeine have been shown to improve lean mass and muscular strength in moderately-trained recreational athletes. Recently, consumption of a supplement before RT that contains protein, caffeine, and creatine has been shown to increase fat-free mass (FFM) and upper-body strength in sedentary, untrained males. Therefore, the purpose of this study was to investigate the impact of pre- and post-workout performance supplementson body composition, circumferences, and maximal strength in resistance trained men.

## Methods

Nine healthy, resistance trained men (age: 24.6± 4.9 years; height: 180.4±5.5cm; weight: 80.7±8.8kg) completed 6 weeks of periodized RT targeting muscles of the arms and shoulders, legs and core, and chest and back with three workouts per week. Resistance increased while repetitions decreased in two-week increments (week 1: 3x10, week 2: 3x6, and week 3: 3x4). Rest intervals of 60-90 seconds were constant between sets. Participants were assigned to one of two groups based upon maximal voluntary contraction of the quadriceps (Biodex) to lean mass ratio. Group 1 (n=6; Performance Supplement; PS) consumed one serving of NO-Shotgun^®^ before each workout and one serving of NO-Synthesize^®^(Vital Pharmaceuticals, Inc., Davie, FL) immediately after each workout and on non-RT days. Group 2 (n= 3; Placebo; PL) consumed a flavor-matched isocaloric maltodextrin placebo in the identical manner. Laboratory measurements included the following: body composition (dual-energy X-ray absorptiometry; DXA), circumferences of the shoulders, chest, waist, hip, and thigh, and maximal strength of the upper (chest press; CP) and lower body (leg press; LP) using one repetition maximum lifts (1RM). Statistical analysis was conducted using a 2x2 repeated measures analysis of variance.Significance was set at p<0.05 and all values are reported as means + standard deviation.

## Results

After 6 weeks, the PS group had a significant increase in FFM (pre, 63.8±6.3 vs. post, 67.1 + 6.7 kg; 5.2 ± 1.4%) with no change in PL group (pre, 66.2 ± 9.1vs. post, 66.9 + 11.3 kg; 0.7± 2.7%). The PS demonstrated a significant increase in CP 1RM (pre, 94.1 ± 16.7 vs. post, 104.1 + 21.5 kg; 7.1 ± 3.6%) with no change in PL (pre, 132.6 ± 16.1 vs. post, 137.9 + 17.4 kg; 9.2 ± 8.3%). There were no group differences in circumferences, except for biceps where PS resulted in a significant (3.2 ± 0.7%) increase compared to the PL group (1.7 ± 2.0%).

## Conclusion

Six weeks of RT and pre- and post-exercise consumption of NO-Shotgun^®^ and NO-Synthesize^®^ improve FFM and chest press 1RM in healthy, resistance-trained men. However, more participants are needed to improve statistical power and support these results.

## Funding

This study was supported by product donation from Vital Pharmaceuticals, Inc., Davie, FL.

